# Human Adenovirus Type 26 Induced IL-6 Gene Expression in an αvβ3 Integrin- and NF-κB-Dependent Manner

**DOI:** 10.3390/v14040672

**Published:** 2022-03-24

**Authors:** Davor Nestić, Ksenija Božinović, Isabela Drašković, Alen Kovačević, Jolien van den Bosch, Jelena Knežević, Jerome Custers, Andreja Ambriović-Ristov, Dragomira Majhen

**Affiliations:** 1 Laboratory for Cell Biology and Signalling, Division of Molecular Biology, Ruđer Bošković Institute, 10000 Zagreb, Croatia; davor.nestic@irb.hr (D.N.); ksenija.bozinovic@irb.hr (K.B.); isabela.pehar@irb.hr (I.D.); akovacevic90@gmail.com (A.K.); jolien.vdbosch@hotmail.com (J.v.d.B.); andreja.ambriovic.ristov@irb.hr (A.A.-R.); 2 Laboratory for Advanced Genomics, Division of Molecular Medicine, Ruđer Bošković Institute, 10000 Zagreb, Croatia; jelena.knezevic@irb.hr; 3 Faculty for Dental Medicine and Health, University of Osijek, 31000 Osijek, Croatia; 4 Janssen Vaccines and Preventions BV, 2333 CA Leiden, The Netherlands; jcuster1@its.jnj.com

**Keywords:** human adenovirus type 26, vaccine vector, innate immune response, integrin αvβ3

## Abstract

The low seroprevalent human adenovirus type 26 (HAdV26)-based vaccine vector was the first adenovirus-based vector to receive marketing authorization from European Commission. HAdV26-based vaccine vectors induce durable humoral and cellular immune responses and, as such, represent a highly valuable tool for fighting infectious diseases. Despite well-described immunogenicity in vivo, the basic biology of HAdV26 still needs some refinement. The aim of this study was to determine the pro-inflammatory cytokine profile of epithelial cells infected with HAdV26 and then investigate the underlying molecular mechanism. The expression of studied genes and proteins was assessed by quantitative polymerase chain reaction, western blot, and enzyme-linked immunosorbent assay. Confocal microscopy was used to visualize HAdV26 cell uptake. We found that HAdV26 infection in human epithelial cells triggers the expression of pro-inflammatory cytokines and chemokines, namely IL-6, IL-8, IL-1β, and TNF-α, with the most pronounced difference shown for IL-6. We investigated the underlying molecular mechanism and observed that HAdV26-induced IL-6 gene expression is αvβ3 integrin dependent and NF-κB mediated. Our findings provide new data regarding pro-inflammatory cytokine and chemokine expression in HAdV26-infected epithelial cells, as well as details concerning HAdV26-induced host signaling pathways. Information obtained within this research increases our current knowledge of HAdV26 basic biology and, as such, can contribute to further development of HAdV26-based vaccine vectors.

## 1. Introduction

Vaccines, biological preparations that provoke specific immunity, are recognized as one of the most cost-effective interventions for the prevention of infectious diseases. A vaccine typically contains an agent that resembles a disease-causing microorganism, and is often made from weakened or killed forms of the microbe, its toxins, or one of its surface antigens. Recombinant adenovirus vectors are excellent shuttles for the delivery of antigens since they mimic natural adenoviral infection and enable its intracellular expression. As a consequence, a potent adjuvant effect can be exerted due to the adenovirus-induced stimulation of various elements of innate and adaptive immunity, causing antigen-specific immune response with protective antibody levels and adequate cellular responses [1].

Human adenoviruses (HAdVs) are non-enveloped double-stranded DNA viruses spanning 67 human serotypes [2,3,4] classified in subgroups A–G. The molecular weight of a functional HAdV particle is about 150 MDa and it has an icosahedral capsid of approximately 90 nm [5]. HAdV infection starts with binding to the primary receptor at the cell surface followed by virus cell entry. HAdVs are highly immunogenic and, following infection, can induce the production of numerous chemokines (macrophage inflammatory protein 1, RANTES, IL-8, and monocyte chemo attractant protein 1) and cytokines (TNF-α, IL-6, IL-1, IL-12, and type I interferons) that modulate the initiation of inflammation and regulate the immune response. Immune responses to HAdV vectors can be induced by the presence of the virus itself, via recognition through pathogen recognition receptors or as a consequence of interactions with cell surface receptors. Signaling triggered by interactions between HAdV fibers and coxsackie adenovirus receptor (CAR) activates the downstream signaling of ERK 1/2, JNK, MAPK, and NF-κB, leading to the upregulation of IL-8, GRO-α, GRO-γ, or RANTES [6]. On the other hand, binding to CD46 and αvβ3/5 integrins leads to Rac1, Pak1, and CtBP1 activation, which can interfere with the innate immunity response [7].

The most common and best-described HAdV so far is human adenovirus type 5 (HAdV5), which is very efficient in terms of transduction efficacy; however, it is disadvantaged by high pre-existing immunity that may limit the efficacy of HAdV-based vectors [8]. Thus, development of new strategies to evade undesired anti-vector–host immune responses is needed, such as the use of vectors based on low seroprevalent human adenoviruses type 26 (HAdV26) or 35 (HAdV35). HAdV26 has emerged as a promising platform for vaccine vector development. Vaccine vectors based on HAdV26 are listed as the intervention in more than 30 clinical trials, among which several are phase III. Very recently, an important milestone in HAdV26-based vaccine vector research was reached when the European Medicines Agency approved two vector vaccines based on HAdV26, namely Ad26.ZEBOV against Ebola and Ad26.COV2.S against COVID-19 [9]. HAdV26-based vaccine vectors have an acceptable safety profile in humans and are able to induce neutralizing [10] and binding antibodies [11], CD4+ and CD8+ T cell responses [12], and a Th1-biased immune response in animals and humans [13]. Vaccination with HAdV26 induced also substantially high levels of antiviral (IFN-γ, IP-10) and pro-inflammatory (IL-1RA, IL-6) cytokines on day 1 following immunization [14].

While HAdV26 immunogenicity in vivo is rather well described and understood, the basic biology of this virus is much less clear. To date, no reports are available describing the host signaling activity induced by HAdV26 infection of epithelial cells. Likewise, there are no studies characterizing the host response to HAdV26 in the context of receptor usage. However, there is evidence that specific immunomodulatory factors can be released after epithelial infection with adenoviruses and that these factors can be involved in virus-induced inflammation [15]. Furthermore, in the context of vaccination, antigens expressed by epithelial cells that receive AdV vector vaccine can be indirectly presented by antigen presenting cells to CD4+ T cells or via cross-presentation to CD8+ T cells [1], indicating a role for epithelial cells in vaccine efficacy. Therefore, in the current study, we determined the pro-inflammatory cytokine profile of epithelial cells infected with HAdV26 and investigated the underlying molecular mechanism. We found that HAdV26 infection of human epithelial cells triggers the expression of pro-inflammatory cytokines, namely IL-6, IL-8, IL-1β, and TNF-α. In order to investigate the possible mechanism underlying HAdV26 induced inflammation, we further explored expression of IL-6 and the corresponding signaling mechanism in relation to HAdV26 infection. By using β3 integrin-specific siRNA we observed that the HAdV26-induced IL-6 gene expression is αvβ3 integrin dependent. We have also shown that IL-6 gene expression triggered by HAdV26 infection is NF-κB mediated. Our findings provide new data regarding pro-inflammatory cytokine expression in HAdV26-infected epithelial cells, as well as details concerning the HAdV26-induced host signaling pathways. Information obtained within this research allows better understanding of HAdV26 basic biology and, as such, can contribute to further development of HAdV26-based vaccine vectors.

## 2. Materials and Methods

*Cells, viruses, antibodies and chemicals.* HEK293 (human embryonic kidney: ATCC CRL-1573), A549 (human lung carcinoma: ATCC CCL-185), and SK-OV-3 (human ovarian carcinoma: ATCC HTB-77) cells were obtained from ATCC Cell Biology Collection and were cultured according to manufacturer’s instructions. Replication-incompetent recombinant adenoviral vectors based on adenovirus type 5, 26, and 35 were previously constructed [16,17]. Viruses were propagated on HEK293 cells and purified by a CsCl gradient. Enhanced green fluorescent protein (eGFP) gene, encoded by adenoviral vector genome and driven by the CMV promoter, served as a reporter gene. Fluorescent labelling of adenoviruses with Alexa Fluor 488 was previously described [18]. Antibodies used for western blot analyses were: p-p38 (Santa Cruz Biotechnology, sc-166182, Dallas, TX, USA); p38 (Santa Cruz Biotechnology, sc-535), pERK (Santa Cruz Biotechnology, sc-7383), ERK (Santa Cruz Biotechnology, sc-94), pJNK (Santa Cruz Biotechnology, sc-6254), JNK (Santa Cruz Biotechnology, sc-7345), IκB-α (Santa Cruz Biotechnology, sc-1643). For inhibiting the nuclear factor-kappa B (NF-kB)-mediated signaling pathway cells were pretreated (1 h, 37 °C) with Bay 11-7082 (Sigma-Aldrich, Burlington, MA, USA) and infected with HAdV26 (10,000 vp/cell) in presence of Bay 11-7082. Final Bay 11-70082 concentrations used were 10 µM for SK-OV-3 and 20 µM for A549. 

*Confocal microscopy.* Intracellular localization of HAdV26 in SK-OV-3 and A549 cells was assessed by confocal microscopy. Cells were seeded in 24-well tissue culture plates on coverslips (2 × 10^4^ cells/well), and 48 h later incubated with Alexa Fluor 488 labelled HAdV26 (10,000 vp/cell) for 1 h at 37 °C. Non-internalized viruses were removed by washing the cells twice with PBS, and cells were fixed with 2% paraformaldehyde in PBS for 12 min at room temperature. Next, cells were permeabilized (0.1% Triton X-100 in PBS for 2 min at room temperature) and incubated with Alexa Fluor 555 Phalloidin (Thermo Fischer Scientific, Waltham, MA, USA) for 15 min at room temperature. Coverslips were mounted in Fluoromount G (Southern Biotech, Birmingham, AL, USA) containing DAPI for nuclei staining. Leica TCS SP8 X inverted confocal microscope (Leica Microsystems, Wetzlar, Germany) with 63x/1.40 oil-immersion objective was used for imaging. The images were analyzed using LAS X (Leica Microsystems, Wetzlar, Germany) software and they show maximum projections of confocal stacks. The qualification of HAdV internalization in cells is presented as the number of virus particles per cell with mean and standard deviations. The N number represents the number of cells analyzed in each sample.

*Reverse transcription quantitative polymerase chain reaction (RT-qPCR).* Gene expression of pro-inflammatory cytokines was assessed by quantitative polymerase chain reaction (qPCR) after reverse transcription (RT) using isolated total RNA. Cells were seeded in 6-well tissue culture plates (3 × 10^5^ cells/well), and 24 h later infected with HAdV (10,000 vp/cell). Cells were collected and total RNA was isolated using High Pure RNA Isolation kit (Roche, Mannheim, Germany) 1 h, 3 h, or 6 h post infection (p.i.). RNA was reverse-transcribed into first strand complementary DNA (cDNA) by a High-Capacity cDNA Reverse Transcription kit (Applied Biosystems, Bedford, MA, USA). cDNA was analyzed by qPCR on a StepOnePlus^TM^ Real-Time PCR System (Thermo Fischer Scientific, Waltham, MA, USA) using Sybr^®^ Green (Applied Biosystems, Bedford, MA, USA). To determe the Ct values, StepOne Software (v 2.3) was used (Applied Biosystems, Bedford, MA, USA). qPCR conditions were: initial denaturation for 10 min at 95 °C, followed by 40 cycles of 15 s at 95 °C and 1 min at the annealing temperature of 60 °C. Housekeeping gene used for assessing ΔΔCt was GAPDH. Fold changes were calculated using the standard 2^−ΔΔCt^ method [19]. PCR primers for analyzed genes are listed in Table 1.

*Enzyme-linked immunosorbent assay (ELISA).* Amounts of secreted cytokines were measured by ELISA. Cells were seeded in 6-well tissue culture plates (3 × 10^5^ cells/well), and, 24 h later, infected with HAdV. Supernatants used for ELISA were collected at 6 h or 24 h p.i. Human IL-6 Uncoated Elisa and Human IL-8 Uncoated Elisa (all Invitrogen, Waltham, MA, USA) were used according to the manufacturer’s instructions.

*Western blot.* Activation of MAP kinases or NF-κB was assessed by western blot. Cells seeded in 6-well tissue culture plates (3 × 10^5^ cells/well) were infected with HAdV and at 1 h or 6 h p.i. lysed with Laemmli buffer (heated to 95 °C), scraped off the plate, sonicated, and boiled (95 °C) for 3 min. Proteins were separated using 10% polyacrylamide gel by SDS-PAGE and transferred to nitrocellulose membrane (Amersham Protran, GE Healthcare, Chicago, IL, USA). Membranes were blocked with 5% non-fat dry milk in Tris-buffered saline containing 1% Tween-20 and probed with specific primary antibodies. Following incubation, blots were visualized with appropriate anti-species adapted horseradish peroxidase-conjugated IgG antibody. Detection was performed with Pierce™ ECL Western Blotting Substrate (Thermo Fisher Scientific, Waltham, MA, USA), while signals were detected using ChemiDoc™ Imaging System (Bio-Rad, Hercules, CA, USA). Densitometry was performed with ImageJ software (J52.A). Proteins were normalized to the corresponding total protein level or total protein level stained with amido black. The results were presented as relative expression or activation of proteins in HAdV infected cells compared to non-infected cells (n.i.).

*siRNA experiments.* To downregulate β3 integrin we used two specific siRNA: Silencer Select Predesigned siRNAs β3 integrin siRNA ID s 7581 (hereafter referred to as si(β3)_1) from Thermo Fisher Scientific and Mission esiRNA human itgb3: EHU051661 from Merck (hereafter referred to as si(β3)_2). Scrambled siRNA #1 (si(-)), catalog No. 4390844, was from Thermo Fisher Scientific. Cells were transfected at a confluency of 70% using Lipofectamine RNAiMAX reagent (Invitrogen, USA) according to the manufacturer’s protocol and used for experiments at 48 h after transfection. Gene expression results assessed by qPCR were presented as relative expression of investigated genes after HAdV infection in cells transfected with si(β3) compared to cells transfected with si(-).

*Flow cytometry.* After β3 integrin downregulation, flow cytometry was used to analyze the expression of αvβ3 integrin. Cells were detached by trypsin and washed twice with PBS; then, 3 × 10^5^ cells/sample were incubated on ice for 1 h with anti-αvβ3 integrin antibody (MAB1976, LM609, Merck Milipore, Darmstadt, Germany) or isotype control (IgG, M5284, Sigma-Aldrich, Burlington, MA, USA). Binding of the unlabeled primary antibody was revealed by incubation with FITC-conjugated anti-mouse IG (554001, BD Biosciences, Franklin Lakes, NJ, USA) as a secondary antibody on ice for 1 h. Analysis was performed on FACSCalibur (BD Biosciences, Franklin Lakes, NJ, USA), and cell acquisition was measured using BD CellQuest software package (BD Biosciences, Franklin Lakes, NJ, USA). Data were analyzed using FCS Express 3 (De Novo Software, Pasadena, CA, USA).

*Statistical analysis.* All experiments were performed at least three times in duplicate or triplicate, except flow cytometry experiments, which were performed twice. All analyses and graphs were created in GraphPad Prism (GraphPad Software Inc., San Diego, CA, USA). Data were analyzed by unpaired Student’s *t*-test, and expressed as mean ± standard deviation (SD). * *p* < 0.05; ** *p* < 0.01; *** *p* < 0.001; **** *p* < 0.0001; ns = non-significant (*p* > 0.05). 

## 3. Results

### 3.1. HAdV26 Infection Triggers Expression of Pro-Inflammatory Cytokines in Human Epithelial Cells In Vitro

Innate cytokine profiles induced in vivo by HAdV26 have been described in detail. It has been demonstrated that in rhesus monkeys, HAdV35, HAdV26, and HAdV48, which are vectors which do not use CAR as their primary receptor, trigger innate cytokine profiles characterized by higher levels of antiviral and pro-inflammatory cytokines than those triggered by HAdV5 vectors [14]. Recently, we reported that HAdV26 uses αvβ3 integrin as a receptor for infecting epithelial cells [18]. The gene expression of pro-inflammatory cytokines induced by HAdV26 infection in epithelial cells has not been previously assessed; therefore, we determined the gene expression of the most common adenovirus induced pro-inflammatory cytokines [20] in A549 and SK-OV-3 cells infected with HAdV26, on the basis that A549 and SK-OV-3 cells differ in expression of αvβ3 integrin and consequently in transduction efficiency. Namely, A549 cells have very low amounts of αvβ3 integrin while SK-OV-3 express αvβ3 integrin, allowing better transduction efficiency with HAdV26 [18]. Of note, both of these cell lines exhibit epithelial characteristics. A549 is an adenocarcinomic human alveolar basal epithelial cell line and SK-OV-3 is a human ovarian cancer cell line with epithelial-like morphology. 

**Figure 1:**
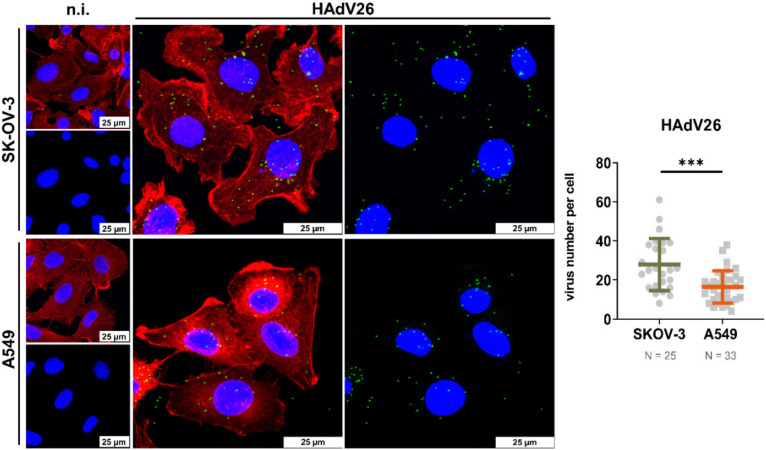
HAdV26 uptake in SK-OV-3 and A549 cells. Cells were incubated with Alexa Fluor 488-labelled HAdV26 (10,000 vp/cell) for 1 h at 37 °C. Non-internalized viruses were rinsed away, and the cells were fixed with 2% paraformaldehyde (PFA). Green, Alexa Fluor 488-labeled virus; blue, nuclei stained with DAPI; red, actin cytoskeleton stained with phalloidin. The images are maximum projections of confocal stacks. Representative confocal images are shown in the left panel. Scale bars = 25 µm. Quantification of virus internalization efficiency, expressed as virus number per cell, is shown in the right panel. The horizontal bars represent means, and the error bars indicate standard deviations; N represents the numbers of cells analyzed, n.i. denotes non-infected, *** *p* < 0.001.

In this study, we measured host gene expression in the early phase of HAdV26 infection, when the resulting signaling will be due only to HAdV26 detection by cellular receptors. In order to determine the cellular uptake of HAdV26 in A549 and SK-OV-3 cells, we fluorescently labelled HAdV26 and observed its localization in studied cells at 1 h p.i. (Figure 1). The average amount of HAdV26 particles per cell was 1.7-fold higher in SK-OV-3 than in A549 cells, indicating that presence of αvβ3 integrin increases HAdV26 cell uptake. 

**Figure 2:**
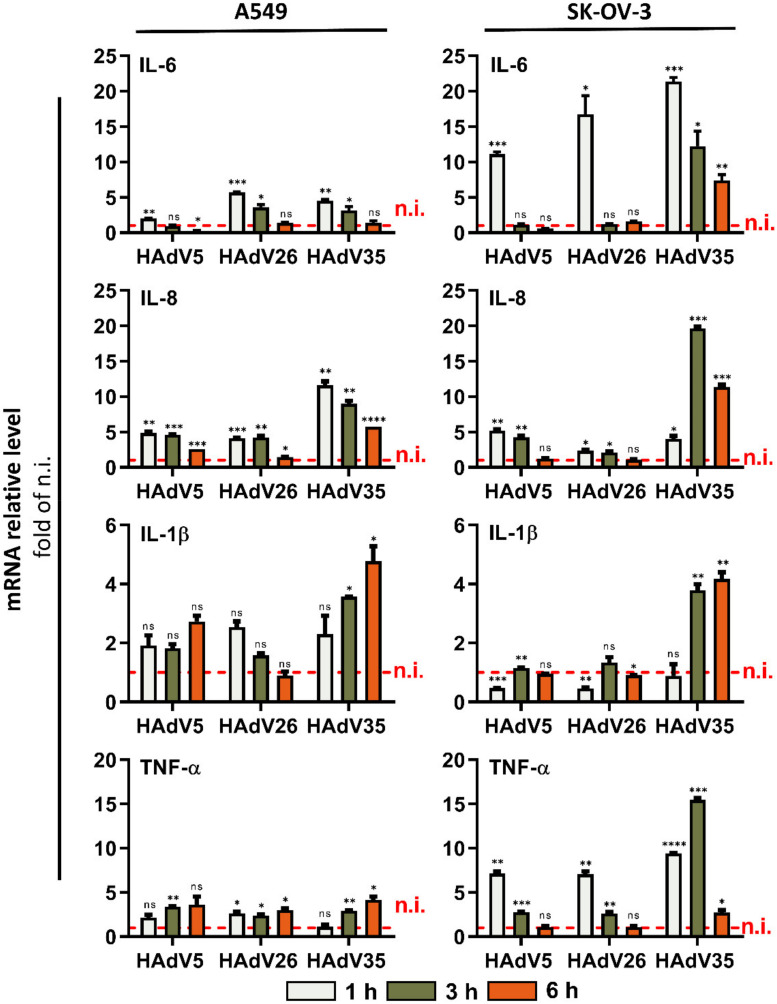
Relative gene expression of pro-inflammatory cytokines in human epithelial cells upon infection with HAdV5, HAdV26, or HAdV35. A549 and SK-OV-3 cells were transduced with 10,000 vp/cell of virus for 1, 3, and 6 h. Relative gene expression was determined by RT-qPCR. Data are shown as mRNA levels relative to the non-infected (n.i.) cells, plus standard deviations. Representative data from three independent experiments yielding similar results are shown (n = 3). * *p* < 0.05; ** *p* < 0.01; *** *p* < 0.001; **** *p* < 0.0001.

We further examined expression of pro-inflammatory cytokines on gene expression level after infection with HAdV26. As benchmark viruses, we used HAdV5 which is so far the best studied adenovirus, and low-seroprevalent HAdV35, used as vaccine vector candidate like HAdV26. As shown in Figure 2, the infection of A549 and SK-OV-3 with HAdV26 induced changes in the expression of the IL-6, IL-8, IL-1β, and TNF-α genes. For all studied genes, the most pronounced change was observed at 1 h p.i. The expression of the IL-8, IL-1β, and TNF-α genes in HAdV26−infected cells is comparable to that in HAdV5-infected cells. Infection with HAdV35 caused increased expression of IL-6, IL-1β, TNF-α, and especially IL-8 in both A549 and SK-OV-3 cells. The most noticeable change in gene expression after infection with HAdV26 was seen for IL-6. Infection with HAdV26 caused a 5.7-fold increase in IL-6 gene expression in A549 cells and a 16.7-fold increase in SK-OV-3 cells at 1 h after infection in comparison to non-infected cells. In addition, the expression of IL-6 after HAdV26 infection in A549 and SK-OV-3 cell lines was higher than that induced by HAdV5 infection. These data indicate that HAdV26 infection of epithelial cells triggers the gene expression of pro-inflammatory cytokines, especially IL-6. Thus, in order to investigate the possible mechanism underlying HAdV26-induced inflammation in the current study, we chose IL-6 as a representative inflammatory effector and further explored its expression in relation to HAdV26 infection. 

### 3.2. HAdV26 Triggered IL-6 Gene Expression Is αvβ3 Integrin Dependent

**Figure 3:**
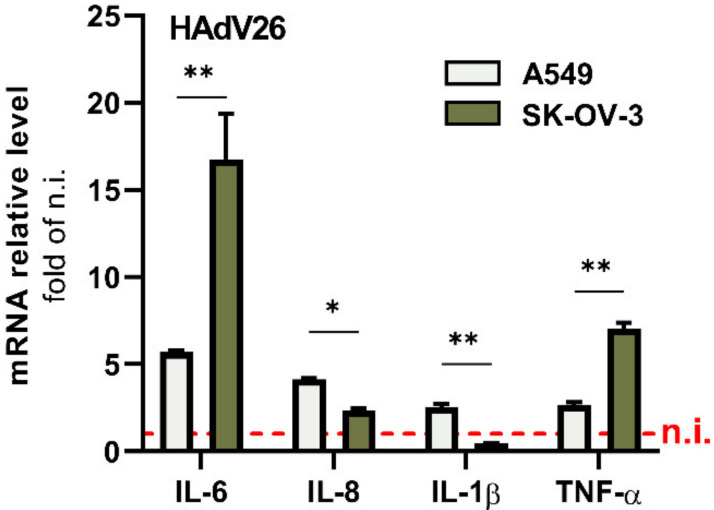
Gene expression of pro-inflammatory cytokines in A549 and SK-OV-3 cells upon infection with 10,000 vp/cell of HAdV26 for 1 h. Data correspond to those shown in Figure 2. * *p* < 0.05; ** *p* < 0.01.

Next, we wanted to examine the differences in the HAdV26-induced gene expression of pro-inflammatory genes between SK-OV-3 and A549 cells in the context of αvβ3 integrin. As can be seen in Figure 3, HAdV26 infection in SK-OV-3 cells resulted in increased expression of IL-6 and moderately increased expression of TNF-α compared to A549 cells. Due to the higher amount of αvβ3 integrin, HAdV26 enters (Figure 1) and infects [18] SK-OV-3 cells more efficiently than A549, which led us to hypothesis that triggering expression of IL-6 and TNF-α might be correlated to the amount of αvβ3 integrin, i.e., the receptor of HAdV26, known for mediating immune responses [21,22]. 

**Figure 4:**
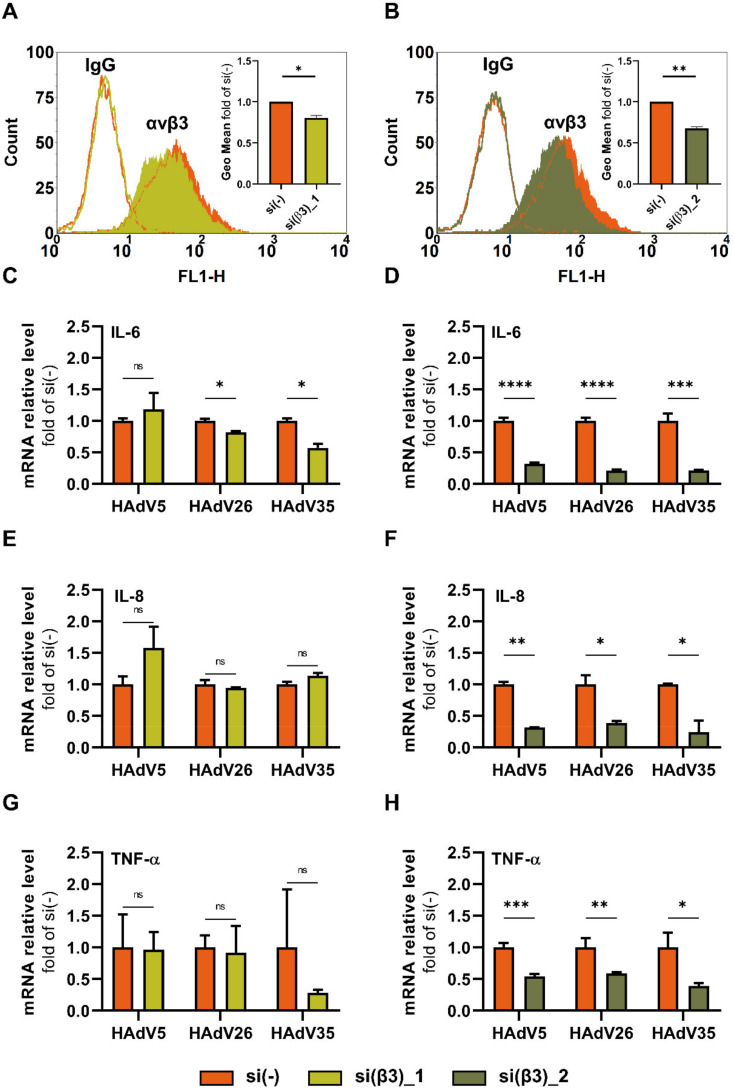
Relative gene expression of IL-6, IL-8, and TNF-α upon infection with HAdV5, HAdV26, or HAdV35 in SK-OV-3 cells with decreased expression of αvβ3 integrin. (**A**,**B**) Expression of αvβ3 integrin in SK-OV-3 cells after downregulating expression of β3 integrin using si(β3)_1 and si(β3)_2, respectively. Thin-line histograms represent isotype controls (IgG) and filled histograms the expression of αvβ3 integrin. Representative data from two independent experiments yielding similar results are shown (n = 2). (**C**,**E**,**G**) Relative gene expression of IL-6, IL-8, and TNF-α upon infection with HAdV5, HAdV26, or HAdV35 in SK-OV-3 cells transfected with si(β3)_1. (**D**,**F**,**H**) Relative gene expression of IL-6, IL-8, and TNF-α upon infection with HAdV5, HadV26, or HAdV35 in SK-OV-3 cells transfected with si(β3)_2. Cells transfected with si(β3)_1 or si(β3)_2 were transduced with 10,000 vp/cell of virus for 1 h. Relative gene expression was determined by RT-qPCR. Data are shown as mRNA levels relative to the cells transfected with scrambled siRNA (si(-)), plus standard deviations. Representative data from three independent experiments yielding similar results are shown (n = 3). * *p* < 0.05; ** *p* < 0.01; *** *p* < 0.001; **** *p* < 0.0001; ns = non-significant (*p* > 0.05).

To further investigate this assumption, we downregulated αvβ3 integrin in SK-OV-3 cells using specific siRNA for the β3 integrin subunit, and subsequently determined the gene expression of IL-6, IL-8 and TNF-α following HAdV26 infection. We used two β3 integrin-specific siRNA, si(β3)_1 and si(β3)_2. Both these integrin-specific siRNAs decreased the expression of αvβ3 integrin on the surface of SK-OV-3 cells (Figure 4A,B). Downregulating αvβ3 integrin using si(β3)_1 decreased the HAdV26-induced expression of the IL-6 gene, but had no influence on the expression of IL-8 or TNF-α (Figure 4C,E,G). Downregulating αvβ3 integrin using si(β3)_2 decreased the HAdV26-induced expression of all three cytokines, namely IL-6, IL-8, and TNF-α (Figure 4D,F,H). Compared to si(β3)_1, si(β3)_2 is composed of a heterogeneous pool of siRNA that all target the same mRNA sequence; thus, the effect is more pronounced both on the level of αvβ3 integrin expression and the expression of the studied cytokines. Data obtained here indicate that the difference in the HAdV26-induced IL-6 profile occurs as the result of enhanced virus uptake in SK-OV-3 cells due to the higher amount of αvβ3 integrin on the cell surface. 

### 3.3. HAdV26-Induced IL-6 Gene Expression Is NF-κB Mediated

**Figure 5:**
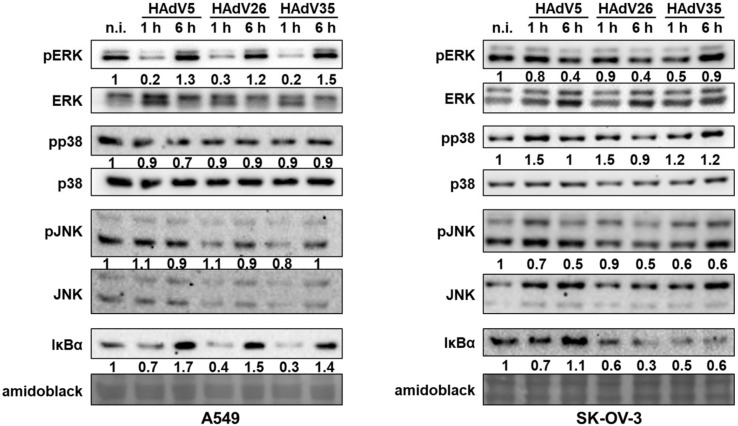
Western blot and densitometric analysis of MAP kinases and NF-κB activation status in HAdV5-, HAdV26-, or HAdV35-infected A549 and SK-OV-3 cells. Cells were transduced with 10,000 vp/cell of virus for 1 h or 6 h. Relative protein amount was determined by western blot. Densitometric analysis is shown between the corresponding protein lines. Proteins were normalized to the corresponding total protein level or total protein level stained with amido black. The results were presented as relative expression or activation of proteins in HAdV-infected cells compared to non-infected cells (n.i.). Amido black staining was also used as control for gel loading. Representative data from three independent experiments yielding similar results are shown (n = 3).

The early host response to adenovirus vectors includes activation of MAP kinases and NF-κB signaling pathways [23]. To examine which signaling pathway is affected after HAdV26 infection, we determined the activation status of the following representative MAP kinases: the extracellular signal-regulated kinases (ERK), the p38 kinases (p38), and the c-Jun NH2-terminal kinases (JNK). In addition, the activation of NF-κB, known to be involved in regulation of cytokine expression, was measured by determining the amount of IκBα, a key factor in NF-κB activation, where signal-induced degradation of IκBα is the effect of NF-κB activation. HAdV-induced host signaling was assessed 1 h and 6 h p.i. The obtained data are shown in Figure 5 and Supplementary Figure S1. We observed a decrease in the activation of ERK in A549 cells at 1 h p.i. with HAdV26. A decrease in ERK activation in SK-OV-3 cells was observed at a later time point, namely 6 h p.i. Infection with HAdV26 had no influence on p38 and JNK activation in A549 cells, but increased p38 activation at 1 h p.i. and decreased JNK activation 6 h p.i. in SK-OV-3 cells. Infection with HAdV26 caused a significant decrease in the amount of IκBα. reflecting the activation of NF-κB signaling. The decrease in IκBα amount in A549 cells was present only at 1 h p.i., but persisted until 6 h p.i. in SK-OV-3 cells. HAdV5 and HAdV35 have a similar pattern in activation status of MAP kinases compared to HAdV26; HAdV35 ERK activation was decreased within 1 h p.i., while for HAdV5 and HAdV26 it decreased only after 6 h p.i. IκBα expression was less reduced after HAdV5 infection, indicating decreased NF-κB activation compared to infection with HAdV26 and HAdV35.

**Figure 6:**
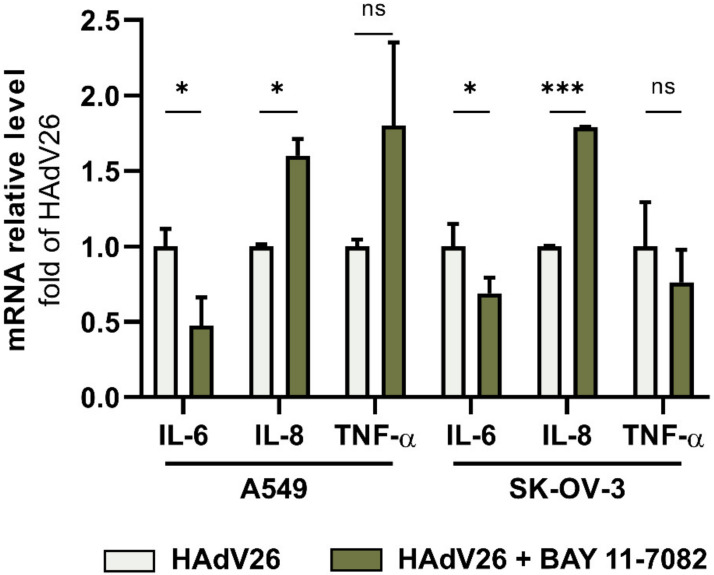
Relative gene expression of IL-6, IL-8, and TNF-α upon infection with HAdV26 in A549 and SK-OV-3 cells treated with NF-κB inhibitor, Bay 11-7082 (10 µM for SK-OV-3, 20 µM for A549). Cells were transduced with 10,000 vp/cell of virus for 1 h. Relative gene expression was determined by qPCR. Data are shown as mRNA levels relative to the cells transfected with scrambled siRNA, plus standard deviations. Representative data from three independent experiments yielding similar results are shown (n = 3). * *p* < 0.05; *** *p* < 0.001; ns = non-significant (*p* > 0.05).

It is known from the literature that NF-κB mediates the induction of pro-inflammatory cytokines such as TNF-α and IL-6 [24]. In order to examine if NF-κB signaling is involved in the HAdV26-induced expression of pro-inflammatory cytokines IL-6, IL-8, and TNF-α, we infected A549 and SK-OV-3 cells with HAdV26 in the presence of an NF-κB inhibitor, Bay 11-7082 [25]. As shown in Figure 6, inhibiting the NF-κB signaling pathway decreased the HAdV26-induced expression of the IL-6 gene by more than 50% in A549 cells and by more than 30% in SK-OV-3 cells. The expression of the IL-8 gene was increased by 50% in both cell lines, while the expression of the TNF-α gene was not affected by the NF-κB inhibitor. Our data suggest that the HAdV26 infection in epithelial cells could increase IL-6 expression through the NF-kB signaling pathway.

### 3.4. HAdV26-Induced Production of IL-6 Is αvβ3 Integrin Dependent

**Figure 7:**
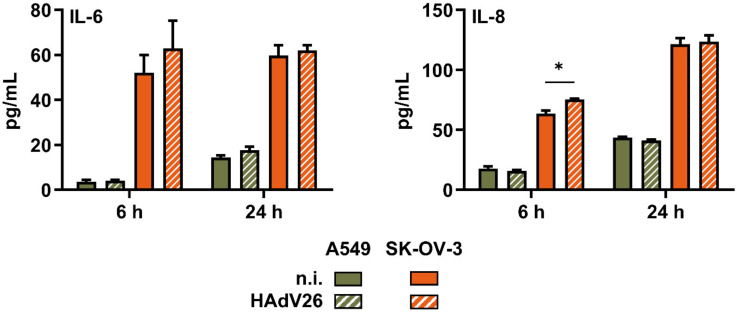
Amount of IL-6 and IL-8 secreted from A549 and SK-OV-3 cells upon infection with 10,000 vp/cell of HAdV26 for 6 h and 24 h. Amount of secreted cytokines was determined by ELISA assay. Data are shown as total amount of secreted protein, plus standard deviations. Representative data from three independent experiments yielding similar results are shown (n = 3). * *p* < 0.05.

Since we observed increased expression of several pro-inflamatory cytokine genes after infecting epithelial cells with HAdV26, we then wanted to examine if this increased gene expression resulted in increased secretion of the corresponding protein. The amount of secreted IL-6 and IL-8 protein was measured at 6 h and 24 h p.i. in A549 and SK-OV-3 cells. As can be seen in Figure 7, a modest increase in IL-6 protein was observed at 6 h p.i. in SK-OV-3 cells and at 24 h p.i. in A549 cells. A meagre increase in IL-8 protein was observed only in SK-OV-3 cells at 6 h p.i. Of note, infecting A549 and SK-OV-3 cells with HAdV35 resulted in significantly increased amounts of IL-6 and IL-8 at both time points p.i. compared to non-infected cells (data not shown). Even though the increase in the IL-6 protein in HAdV26-infected SK-OV-3 cells at 6 h p.i. is moderate, it can be correlated with a higher amount of αvβ3 integrin in these cells, since we did not observe the same in A549 cells. 

**Figure 8:**
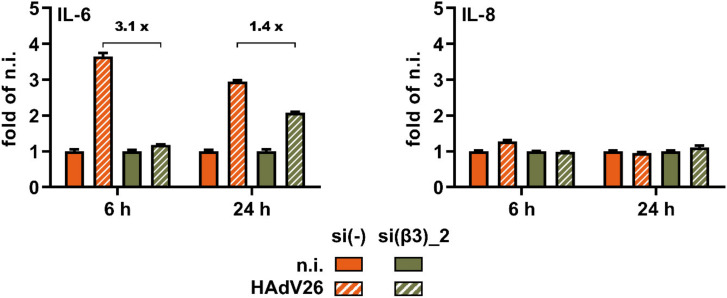
Amount of IL-6 and IL-8 secreted from SK-OV-3 cells with decreased expression of αvβ3 integrin upon infection with 10,000 vp/cell of HAdV26 for 6 h and 24 h. Amount of secreted cytokines was determined by ELISA assay. Data are shown as fold change of total amount of secreted protein in the non-infected (n.i.) cells, plus standard deviations. Representative data from three independent experiments yielding similar results are shown (n = 2).

In order to confirm the role of αvβ3 integrin in the production of IL-6 and IL-8 after infection with HAdV26 we measured the amounts of those cytokines at 6 h and 24 h p.i. in SK-OV-3 cells transfected with β3 integrin-specific siRNA, si(β3)_2. The amount of secreted IL-6 in HAdV26-infected SK-OV-3 cells with decreased expression of αvβ3 integrin was significantly lower than that in the control cells. At 6 h p.i., the amount of IL-6 secreted 6 was 3.1 times lower in the cells with decreased expression of αvβ3 integrin compared to cells transfected with si(-), and 1.4 times lower at 24 h p.i. The amount of IL-8 secreted in HAdV26-infected SK-OV-3 cells with decreased expression of αvβ3 integrin was comparable to the amount secreted from the control cells (Figure 8). 

## 4. Discussion

Cell types that can receive AdV vector following intramuscular vaccination include antigen presenting cells, dendritic cells, muscle cells, fibroblasts, and epithelial cells [26]. The antigen encoded by the AdV-based vaccine expressed by infected muscle or epithelial cell can be presented by antigen presenting cell to CD4+ T cells or via cross-presentation to CD8+ T cells [1], thus influencing vaccine efficacy. Host immune system can detect adenovirus capsid, DNA, or infection itself during almost all steps of the adenovirus infection pathway, from binding and endocytosis to intracellular trafficking. The initial phase of infection, receptor binding and cell uptake, can trigger the expression of pro-inflammatory cytokines that can subsequently influence all cells in the surroundings. Epithelial cells possess pattern recognition receptors that enable them to sense and respond to incoming pathogens, thus triggering pro-inflammatory signals soon after virus interactions. It has been reported that the co-culture of mouse lung epithelial cells and macrophages synergistically augmented its inflammatory responses to AdV infection compared to the epithelial cells or macrophages alone, indicating that the interactions between these two cell types are critical for the AdV-induced innate immune response [27]. Anti-vector adaptive immune response, i.e., neutralizing antibodies against vector itself, may limit the efficacy of a viral vector; hence, the potential role of epithelial cells in initiating the innate immune response to adenoviral vectors deserves careful investigation [28].

In this study, we investigated expression of pro-inflammatory cytokine genes in epithelial cells infected with HAdV26 and found that HAdV26 infection of human epithelial cells triggers the expression of pro-inflammatory cytokines, namely IL-6, IL-8, IL-1β, and TNF-α. By further exploring the mechanism responsible for HAdV26-induced IL-6 expression, we observed that it was αvβ3 integrin dependent and NF-κB mediated. To the best of our knowledge, this is the only study describing early host signaling in HAdV26-infected cells.

Here, we determined the expression of pro-inflammatory genes in A549 and SK-OV-3 cells infected by HAdV26. The infection of A549 and SK-OV-3 with HAdV26 induced changes in the expression of the IL-6, IL-8, IL-1β, and TNF-α genes; namely the expression of all four genes was increased in HAdV26-infected A549 cells, while HAdV26 infection resulted in increased expression of IL-6 and TNF-α in SK-OV-3 cells. The most pronounced increase after infection with HAdV26 was observed for IL-6, whose expression was increased 6 times in A549 cells and more than 15 times in SK-OV-3 cells. Our results are in line with those from the literature, where the increased expression of IL-6 was reported in BEAS-2B cells, a human bronchial epithelial cell line, after infection with HAdV3 and HAdV7 [29]**,** and in primary human bronchial epithelial cells infected with HAdV-B14p1 [30]. With regard to the expression of IL-8, increased expression of this cytokine was reported in A549 cells infected with HAdV7 [31,32] and human corneal fibroblasts [33] and keratinocytes [34] infected with HAdV19. It is necessary to emphasize that the internalization of HAdV26 is significantly decreased compared to that of HAdV5 and HAdV35 in both A549 and SK-OV-3 cells [18], but that it results in greater expression of pro-inflammatory genes than HAdV5 and comparable expression to HAdV35.

Previously, we demonstrated that due to higher expression of αvβ3 integrin, HAdV26 infects SK-OV-3 cells more efficiently than A549 cells [18]. Here, we presented that HAdV26 cell uptake is also increased in cells with higher expression of αvβ3 integrin; thus, we hypothesized that there might also be differences in the HAdV26-induced innate immune response between these two cell lines. We compared the expression of IL-6, IL-8, IL-1β, and TNF-α between HAdV26-infected A549 and SK-OV-3 cells and observed the biggest difference in the expression of IL-6, for which we saw a three-fold increase in expression in SK-OV-3 cells compared to A549 cells. In order to investigate role of this receptor in the HAdV26-induced expression of IL-6 and TNF-α, we downregulated the amount of αvβ3 integrin by using specific siRNA in SK-OV-3 cells prior to HAdV26 infection. Downregulating αvβ3 integrin decreased the HAdV26-induced expression of IL-6, IL-8, and TNF-α genes, but had only a significant influence only on the production of IL-6. Namely, the decreased expression of αvβ3 integrin caused a decrease in the secretion of IL-6 after infection with HAdV26. The role of integrins in mediating the HAdV-induced production of pro-inflammatory cytokines and chemokines was studied in macrophages, where it was shown that in response to HAdV5, macrophage-derived IL-1a triggered the IL-1RI-dependent production of a defined set of pro-inflammatory cytokines and chemokines. The IL-1a-mediated response required the selective interaction of virus RGD motifs with macrophage β3 integrins [35]. Comparable results were obtained in vivo by using a model of integrin β3−/− mice in which it was shown that cytokine induction by oncolytic adenovirus was β3 integrin dependent. Namely, adenovirus-induced cytokines (IL-1β, IL-6, and TNF-α) were significantly higher in the circulation of WT compared with β3−/− animals [36]. The latter data regarding IL-6 are in good agreement with our results indicating that HAdV-induced IL-6 expression and production are β3 integrin dependent both in epithelial cells in vitro and in animal models. Besides HAdV, αvβ3-integrin is involved in recognizing another DNA virus, herpes simplex virus-1, i.e., αvβ3-integrin has been reported as a major sensor and activator of innate immunity to herpes simplex virus-1 [21].

Adenovirus infection can activate host cell signaling, which most commonly involves the MAP kinase pathway. Here, we explored if HAdV26 infection triggers early events in the signal transduction pathways that induce the synthesis of cytokines and thus contribute to the inflammatory response. We therefore determined activation status of ERK, p38, and JNK in epithelial cells following infection with HAdV26. We saw that HAdV26 infection decreased ERK phosphorylation but had no or minor influence on the activation of p38 or JNK. However, we did observe increased activation of NF-κB, which was also required for increased expression of the IL-6 gene. The role of MAP kinases in pro-inflammatory cytokine induction due to the adenovirus infection was reported for different HAdV serotypes. It has been shown that the Ras/Raf/MEK/ERK pathway is necessary for the induction of IL-8 by HAdV7 [32], while this same virus triggers the expression of IL-6 in human airway epithelial cells via the p38/NF-κB signaling pathway [37]**,** which was in line with our results. NF-κB activation was equally important for the expression of the chemokine CXCL10 induced by HAdV5 infection in kidney epithelium-derived cells [38]. Interestingly, NF-κB was also found to mediate the leaky expression of adenovirus genes from the HAdV5 vector genome and the inhibition of NF-κB leads to the suppression of HAdV5 gene expression and hepatotoxicity following transduction with HAdV5-based vectors [39]. In this study, NF-κB inhibition decreased HAdV26-induced IL-6 expression, which could potentially correlate to decreased toxicity. Correlation between NF-κB and IL-6 was also reported in the case of Kaposi’s sarcoma herpesvirus infection. MicroRNA miR-K12-1, encoded by this virus, activates transcription factor STAT3 indirectly through inducing NF-κB activation and NF-κB-dependent expression of IL-6 by repressing the expression of the NF-κB inhibitor IκBα [40]. Similarly, activation of the transcription factor NF-κB, involving the small GTPase Rac1, was required for IL-6 production and subsequent STAT3 activation in human papillomavirus infection [41], indicating that link between NF-κB and IL-6 might be a common feature of DNA viruses.

## 5. Conclusions

Even though vaccine vectors can benefit from the activation of host innate immune response, undesired inflammation can limit their efficacy. Thus, it is important to understand the host response to HAdV vectors in order to further optimize and customize vaccine vector design aimed at specific diseases. Our findings presented in this study may provide a mechanistic link between HAdV26–αvβ3 integrin interactions and the activation of the NF-κB signaling pathway that triggers IL-6 expression, i.e., innate immune and inflammatory responses, and represents an expansion of the current knowledge of HAdV26 biology.

## Figures and Tables

**Table 1:** Primer sequences for qPCR analysis of IL-6, IL-8, IL-1β, and TNF-α gene expression.

Gene ID	Primer Sequences
IL-6	**F:** ^5′^CAATGAGGAGACTTGCCTGG^3′^ **R:** ^5′^GCACAGCTCTGGCTTGTTCC^3′^
IL-8	**F:** ^5′^GTTTTTGAAGAGGGCTGAGAATTC^3′^ **R:** ^5′^ATGAAGTGTTGAAGTAGATTTGCTTG^3′^
IL-1β	**F:** ^5′^TGGCAATGAGGATGACTTGTTC^3′^ **R:** ^5′^CTGTAGTGGTGGTCGGAGATT^3′^
TNF-α	**F:** ^5′^AACCTCCTCTCTGCCATCAA^3′^ **R:** ^5′^GGAAGACCCCTCCCAGATAG^3′^

## Data Availability

Data is contained within the article.

## Supplementary Materials

The following supporting information can be downloaded at: https://www.mdpi.com/article/10.3390/v14040672/s1, Figure S1: Quantification of the protein bands obtained by densitometric analysis from at least three independent Western blot experiments. Data are presented as means ± SD compared to non-infected cells. * *p* < 0.05; ** *p* < 0.01; *** *p* < 0.001; **** *p* < 0.0001; ns = non-significant (*p* > 0.05).
