# Supplementary material for: Human Adenovirus Type 26 Induced IL-6 Gene Expression in an αvβ3 Integrin- and NF-κB-Dependent Manner
Source: Viruses. 2022 Mar 24;14(4):672. doi: 10.3390/v14040672 (PMC9028149; doi:10.3390/v14040672)
Supplement: Supplementary file 1 [file viruses-14-00672-s001.zip › viruses-1614626-supplementary.pdf]

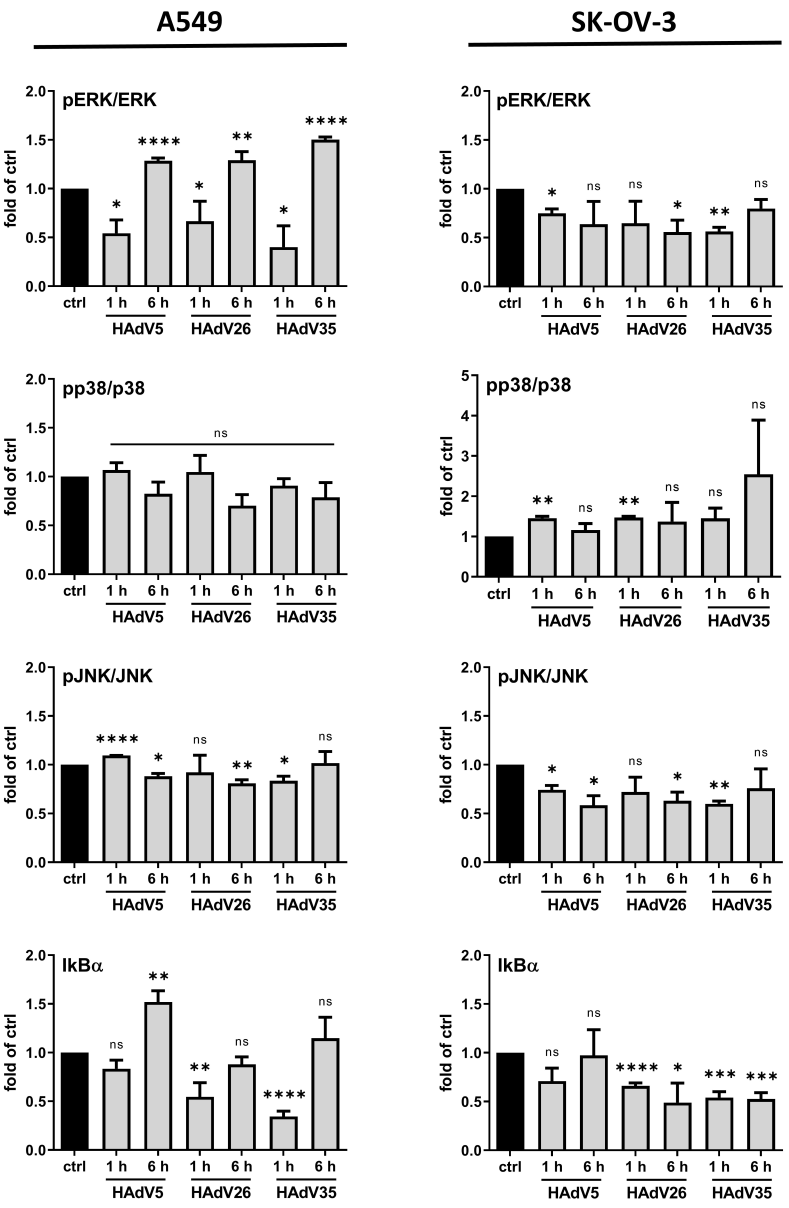


Figure S1: Quantification of the protein bands obtained by densitometric analysis from at least three independent Western blot experiments. Data are presented as means ± SD compared to non-infected cells.* $p < 0.05$; ** $p < 0.01$; *** $p < 0.001$; **** $p < 0.0001$; ns = non-significant ($p > 0.05$).
